# Dexmedetomidine Inhibits ASIC Activity *via* Activation of α_2A_ Adrenergic Receptors in Rat Dorsal Root Ganglion Neurons

**DOI:** 10.3389/fphar.2021.685460

**Published:** 2021-05-24

**Authors:** Shuang Wei, Chun-Yu Qiu, Ying Jin, Ting-Ting Liu, Wang-Ping Hu

**Affiliations:** ^1^ Research Center of Basic Medical Sciences, School of Basic Medical Sciences, Hubei University of Science and Technology, Xianning, China; ^2^ Department of Pharmacology, Hubei University of Science and Technology, Xianning, China

**Keywords:** dexmedetomidine, α2 adrenergic receptor, acid-sensing ion channel, electrophysiology, dorsal root ganglion neuron, nociceptive behavior

## Abstract

Dexmedetomidine (DEX), a selective α_2_ adrenergic receptor (α_2_-AR) agonist, has been shown to have peripheral analgesic effects in a variety of pain conditions. However, the precise molecular mechanisms have not yet been fully elucidated. Acid sensing ion channels (ASICs) are the major player in pain associated with tissue acidosis. Given that both α_2_-ARs and ASICs exist in dorsal root ganglia (DRG) neurons, we therefore investigated the effects of DEX on the functional activity of ASICs. Herein, whole-cell patch-clamp recordings demonstrated that DEX suppressed ASIC-mediated and acid-evoked currents and action potentials in dissociated rat DRG neurons. DEX shifted downwards concentration-response curve to protons, with a decrease of 35.83 ± 3.91% in the maximal current response to pH 4.5. DEX-induced inhibition of ASIC currents was blocked by the α_2A_-AR antagonist BRL44408 in DRG neurons. DEX also inhibited ASIC3 currents in CHO cells co-expressing ASIC3 and α_2A_-ARs, but not in ASIC3 transfected CHO cells without α_2A_-ARs expression. DEX-induced inhibition of ASIC currents was mimicked by the protein kinase A inhibitor H-89, and blocked by intracellular application of the G_i/o_ protein inhibitor pertussis toxin and the cAMP analog 8-Br-cAMP. In addition, peripherally administration of DEX dose-dependently relieved nociceptive responses to intraplantar injection of acetic acid in rats through local α_2A_-ARs. Our results indicated that DEX inhibited the functional activity of ASICs via α_2A_-ARs and intracellular G_i/o_ proteins and cAMP/protein kinase A signaling pathway in rat DRG neurons, which was a novel potential mechanism that probably mediated peripheral analgesia of DEX.

## Introduction

Dexmedetomidine (DEX) is a potent highly selective α_2_ adrenergic receptor (α_2_-AR) agonist. It is approved as an analgesic agent since the early 1970s (Kamibayashi and Maze, 2000). DEX’ action is related to the wide distribution of α_2_-ARs in the pain signaling pathway, including in primary afferents and spinal dorsal horn (Gold et al., 1997; Stone et al., 1998; Shi et al., 2000; Nicholson et al., 2005). DEX has shown potential analgesic effects at the supraspinal, spinal and peripheral levels in various pain conditions. Intrathecal or systemic administration of DEX produces analgesic effects in animals and humans (Aho et al., 1991; Kingery et al., 2000; Kimura et al., 2012). Peripheral DEX also shows anti-nociceptive effects since peripherally restricted α_2_-AR antagonist could block the effect of systemic DEX on neuropathic pain (Poree et al., 1998; Lee et al., 2013). Moreover, DEX has stronger analgesic effects following peripheral nerve injury via α_2_-ARs (Poree et al., 1998; Malmberg et al., 2001). Studies with α_2_-AR knock-out animal models suggest that α_2_-AR plays a major role in DEX analgesia (Hunter et al., 1997; Stone et al., 1997; Malmberg et al., 2001). In addition to neuropathic pain, studies have also confirmed that DEX has analgesic effects on pain under postoperative, acute and chronic inflammatory conditions (Mahmoud and Mason, 2015; Zhao et al., 2020). Clinically, DEX has been used to relieve acute postoperative pain, ischemic pain, refractory cancer pain (Hoy and Keating, 2011).

Apart from α_2_-ARs, a number of ligand-gated and voltage-gated ion channels are also expressed in primary sensory neurons, including dorsal root ganglion (DRG) neurons, which can convert noxious stimulation into ascending nociceptive signals (Mccleskey and Gold, 1999). Thus, modulation of these ion channels may be a potential mechanism underlying peripheral anti-nociceptive effects of DEX. For example, DEX has been shown to inhibit voltage-gated sodium channels via α_2_-ARs in DRG and trigeminal ganglion neurons (Gu et al., 2015; Im et al., 2018). DEX also inhibits the activity of TRPV1 in DRG neurons depending on the activation of α_2_-ARs followed by the inhibition of the adenylate cyclase/cAMP/protein kinase A (PKA) pathway (Lee et al., 2020).

To detect changes in pH, primary sensory neurons also express several acid sensors, such as acid sensitive ion channels (ASICs), TRPV1, proton-sensing GPCRs, and certain K2P channels (Holzer, 2009; Pattison et al., 2019). Among these, ASICs and TRPV1 have been most thoroughly studied. ASICs, as pH sensors, are expressed in both DRG cell bodies and sensory terminals, where they contribute to proton-evoked nociceptive signaling (Alvarez De La Rosa et al., 2002; Benson et al., 2002). To date, there are seven different ASIC subunits encoded by five different genes (Wemmie et al., 2013). Among these ASIC subunits, ASIC3 subunit is the most abundant in DRG and has emerged as a critical pH sensor (Deval et al., 2008; Lee et al., 2013). Proton, a canonical ligand for ASICs, is released and causes tissue acidosis under multiple pathological conditions such as inflammation, tissue injury, ischemic stroke and cancer (Deval et al., 2011; Deval and Lingueglia, 2015). Amiloride, a non-selective ASIC inhibitor, can significantly alleviate the pain caused by moderate (up to pH 6.0) pH, suggesting that ASICs, rather than TRPV1 or other acid sensors, mainly mediate the pain sensation (Ugawa et al., 2002; Deval et al., 2008). ASICs, especially ASIC3, are the major player in pain associated with tissue acidosis and represent novel potential targets for development of analgesics (Reeh and Steen, 1996; Wemmie et al., 2013; Li and Xu, 2015; Dulai et al., 2020).

Given that both α_2_-ARs and ASICs exist in DRG neurons, the aim of this study was to investigate whether ASICs are also modulatory targets of DEX. We observed that DEX inhibited the electrophysiological activity of ASICs through activation of α_2_-ARs and an intracellular cAMP and PKA signaling pathway in rat DRG neurons. DEX also relieved ASIC-mediated nociceptive behaviors in rats by activating peripheral α_2_-ARs.

## Materials and Methods

### Preparation of DRG Neurons

All experimental protocols were approved by the animal research ethics committee of Hubei University of Science and Technology. Sprague-Dawley male rats (5–6 weeks old) were anesthetized and then killed. The DRGs were removed and chopped with thin spring scissors. The minced ganglia were transferred to a test tube containing Dulbecco’s modified Eagle’s medium (DMEM, Sigma) and incubated in a shaking for 25–30 min at 35°C. Incubation solution contained 1.0 mg/ml collagenase (type I-A, Sigma), 0.5 mg/ml trypsin (type II-S, Sigma) and 0.1 mg/ml DNase (type IV, Sigma). Trypsin digestion was terminated by adding1.25 mg/ml Soybean trypsin inhibitor (type II-S, Sigma). The cells were cultured in DMEM supplemented with 10% fetal bovine serum and 100 ng/ml never growth factor (NGF) for 12–24 h at 37°C in a water saturated atmosphere with 95% O_2_ and 5% CO_2_.

### Electrophysiological Recordings

Electrophysiological experiments were carried out as described previously (Wei et al., 2021). Whole-cell patch clamp recordings were carried out at room temperature (22–25°C) using EPC-10 patch clamp amplifier and PULSE software (HEKA Electronic, Lambrecht, Germany). The isolated DRG neurons were transferred to a 35 mm culture dish and kept in normal external solution for at least 60 min before electrophysiological recordings. The external solution contained the following (in mM): 150 NaCl, 5 KCl, 2 MgCl_2_, 2.5 CaCl_2_, 10 HEPES, 10 d-glucose. Its pH and osmolarity was adjusted to 7.4 with NaOH and 330 mOsm/L with sucrose, separately. Recording pipettes were pulled using a Sutter P-97 puller (Sutter Instruments, CA, United States) and its resistance was in the range of 3–6 MΩ. The micropipettes solution contained (in mM): 140 KCl, 2 MgCl_2_, 11 EGTA, 10 HEPES, 4 ATP, and 0.3 Na_2_GTP. Its pH and osmolarity was adjusted to 7.2 with KOH and 310 mOsm/L with sucrose, separately. After whole-cell configuration established, 70–80% series resistance and membrane capacitance current were compensated. The recording currents were sampled at 10 kHz and filtered at 2 kHz. Data detection and analysis were performed using the pCLAMP 10 software (Axon Instruments, CA, United States). The neurons selected for electrophysiological recordings were 15–35 μm in diameter, which are thought to be nociceptive neurons. The membrane voltage was maintained at −60 mV in all voltage-clamp experiments. Current-clamp recordings were obtained by switching to current-clamp mode after a stable whole-cell configuration was formed in voltage-clamp mode. Only cells with a stable resting membrane potential (more negative than −50 mV) were used in the study.

### Cell Culture and Transfection

Rat ASIC3 and human α_2A_-AR cDNAs were used for heterologous expression in Chinese hamster ovary (CHO) cells as described previously (Wu et al., 2017). In brief, CHO cells were cultured at 37°C in a humidified atmosphere of 5% CO_2_ and 95% O_2_ and passaged twice a week. Transient transfection of CHO cells was performed using a HilyMax liposome transfection reagent (Dojindo Laboratories). CHO cells were maintained in F-12 nutrient mixture (added 1.176 g of NaHCO_3_/L medium) supplemented with 10% fetal bovine serum and 1% gluta-MAXTM-1 (×100; Invitrogen). When ASIC3 and α_2A_-AR cDNA were co-transfected, the ratio was maintained at 1:1. All plasmids contained, in addition to the desired ASIC3 cDNA, the coding sequence for enhanced green fluorescent protein to aid in the identification of transfected cells. Electrophysiological measurements were performed 24–48 h after transfection.

### Drug Application

In the experiment, drugs included hydrochloric acid, dexmedetomidine, clonidine, yohimbine, BRL44408, 8-Br-cAMP, H-89, amiloride, APETx2, capsaicin, and AMG 9810. They were obtained from Sigma Chemical Co. (St. Louis, MO, United States). Different pH values were configured with hydrochloric acid and external solution. The working concentration of drugs was freshly prepared in normal external solution and their pH was adjusted to7.4 with NaOH. Each working drug was stored in a series of independent reservoirs and applied by gravity. The distance was ∼30 μm between drug exit and recorded neurons. To block G-protein and intracellular signal, some antagonists or blockers were dissolved in the internal solution and applied for intracellular dialysis through recording patch pipettes as described previously (Liu et al., 2021). To ensure that dialysis drugs are infused into the cell interior, there is at least a 30 min interval between the establishment of the whole cell pathway and the current measurement. To functionally characterize ASIC activity, we used AMG9810 (5 μM) to block TRPV1 in the extracellular solution.

### Nociceptive Behavior Induced by Acetic Acid in Rats

Behavior experiments were carried out as described previously (Wei et al., 2021). Male rats were allowed to habituate for at least 30 min before nociceptive behavior experiments in a Plexiglas chamber. Separate groups of rats were coded and pretreated with 50 μl AMG 9810 (10 μM) together with vehicle, different dose (10, 30 and 100 ng) of DEX, 150 ng BRL44408 + 100 ng DEX in ipsilateral hindpaw using a 30-gauge needle connected to a 100 μl Hamilton syringe. After 5 min, another experimenter subcutaneously administered acetic acid solution (1%, 50 μl) into the hindpaw and tested nociceptive behavior. In one group, acetic acid was injected into one hindpaw and DEX (100 ng) was injected into contralateral hindpaw. Nociceptive behavior (that is, number of flinches) was counted over a 5 min period starting immediately after the injection (Deval et al., 2008; Omori et al., 2008).

### Data Analysis

Data were statistically compared using the Student's t-test or one-way analysis of variance (ANOVA), followed by Bonferroni’s post hoc test. Statistical analysis of concentration–response data was performed using nonlinear curve-fitting program ALLFIT. Data are expressed as mean ± S.E.M.

## Results

### DEX Inhibits ASIC Currents in Rat DRG Neurons

**FIGURE 1:**
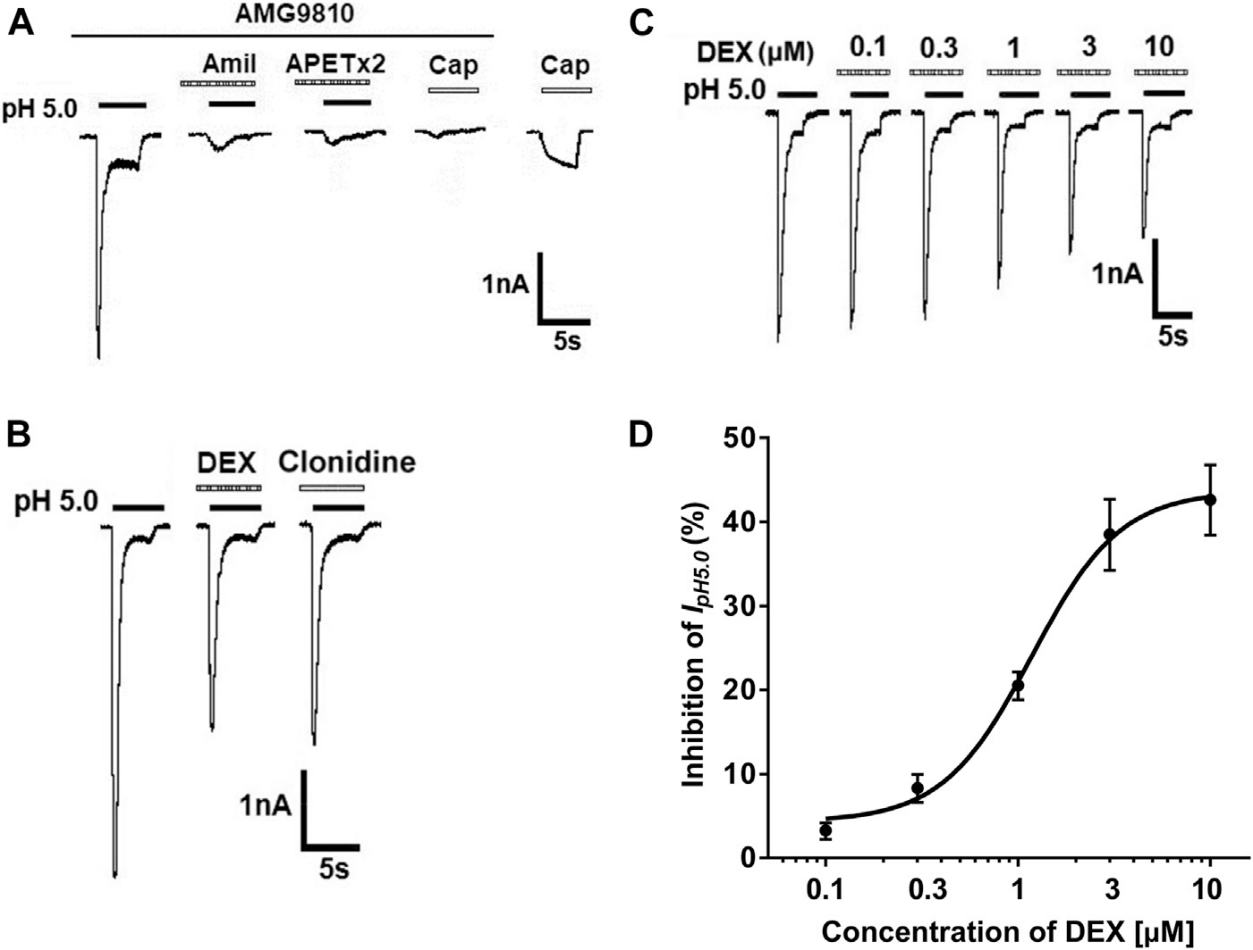
DEX concentration-dependently inhibits proton-gated currents in rat DRG neurons. **(A)**. Representative current traces were evoked by application of a pH 5.0 acidic solution for 5 s in a tested DRG neuron in the presence of AMG9810 (5-μM). The proton-gated current (I_pH5.0_) could be blocked by broad-spectrum ASIC channel blocker amiloride (Amil, 10 μM) and ASIC3 blocker APETx2 (2 μM). Capsaicin (Cap, 100 nM) failed to evoke any membrane currents in the presence of AMG9810 (5-μM). However, capsaicin (100 nM) evoked an inward current after washout of AMG9810 in the same neuron. All membrane potentials were clamped at −60 mV. **(B)**. In the same DRG cell, pre-application of α_2_-ARagonist DEX or clonidine caused an inhibitory effect on I_pH5.0_. **(C)**. The sequential current traces illustrate that I_pH5.0_ amplitude was inhibited by pre-application of different concentration of DEX (for 2 min) in a representative DRG neuron. **(D)**. The graph shows concentration-effect curve of DEX on I_pH5.0_ with an IC_50_ of 1.18 ± 0.11 μM. Each point represents the mean ± SEM of 6–10 cells.

In the majority of DRG neurons tested (76.9%, 10/13), a perfusion of pH 5.0 acid solution to DRG neurons for 5 s evoked a rapid inward current (I_pH5.0_), even though blockade of proton-induced TRPV1 activation by addition of AMG9810 (5 μM) in external solution (Figure 1A). Most (70.0%, 7/10) of I_pH5.0_ were characterized by a fast inactivated inward current, followed by a smaller and non-desensitizing sustained current in ten DRG neurons sensitive to pH 5.0 acid stimuli. All seven I_pH5.0_ currents with this characteristic could be completely blocked by the broad-spectrum ASIC channel blocker amiloride (10 μM) and ASIC3 blocker APETx2 (2 μM). In the presence of AMG9810, capsaicin (100 nM) failed to evoke any membrane currents in all DRG neurons tested. However, capsaicin (100 nM) evoked an inward current in the majority of DRG neurons sensitive to pH 5.0 acid stimuli (71.4%, 5/7) after washout of AMG9810. Thus, these proton-induced currents were considered to be ASIC currents or ASIC3 currents after TRPV1 activation was blocked by AMG9810. In the following study, we mainly observed the ASIC3-like currents with the above characteristics.

In some DRG neurons sensitive to pH 5.0 acid stimuli (52.9%, 9/17), pre-application of α_2_-AR agonist DEX (3 μM) for 2 min decreased the peak amplitude of the ASIC currents (Figure 1B). In all nine cells response to DEX, pre-application of clonidine (10 μM) for 2 min had also similar inhibitory effects on the ASIC currents (Figure 1B). In addition, DEX shortened the inactivation time constant of ASICs from 1,413.81 ± 184.64 to 1,094.44 ± 146.38 msec (*p* < 0.05, paired *t*-test, n = 9). The inhibition of I_pH5.0_ was dependent upon the concentration of DEX (Figures 1C,D). In a representative DRG neuron, the peak amplitude of I_pH5.0_ progressively decreased as concentration of pre-treated DEX increased from 0.1 to 10 μM (Figure 1B). Figure 1C shows concentration-effect curve of DEX on I_pH5.0_ with an IC_50_ (half-maximal effective concentration) value of 1.18 ± 0.11 μM. The results indicated that DEX concentration-dependently inhibited ASIC currents in rat DRG neurons.

**FIGURE 2:**
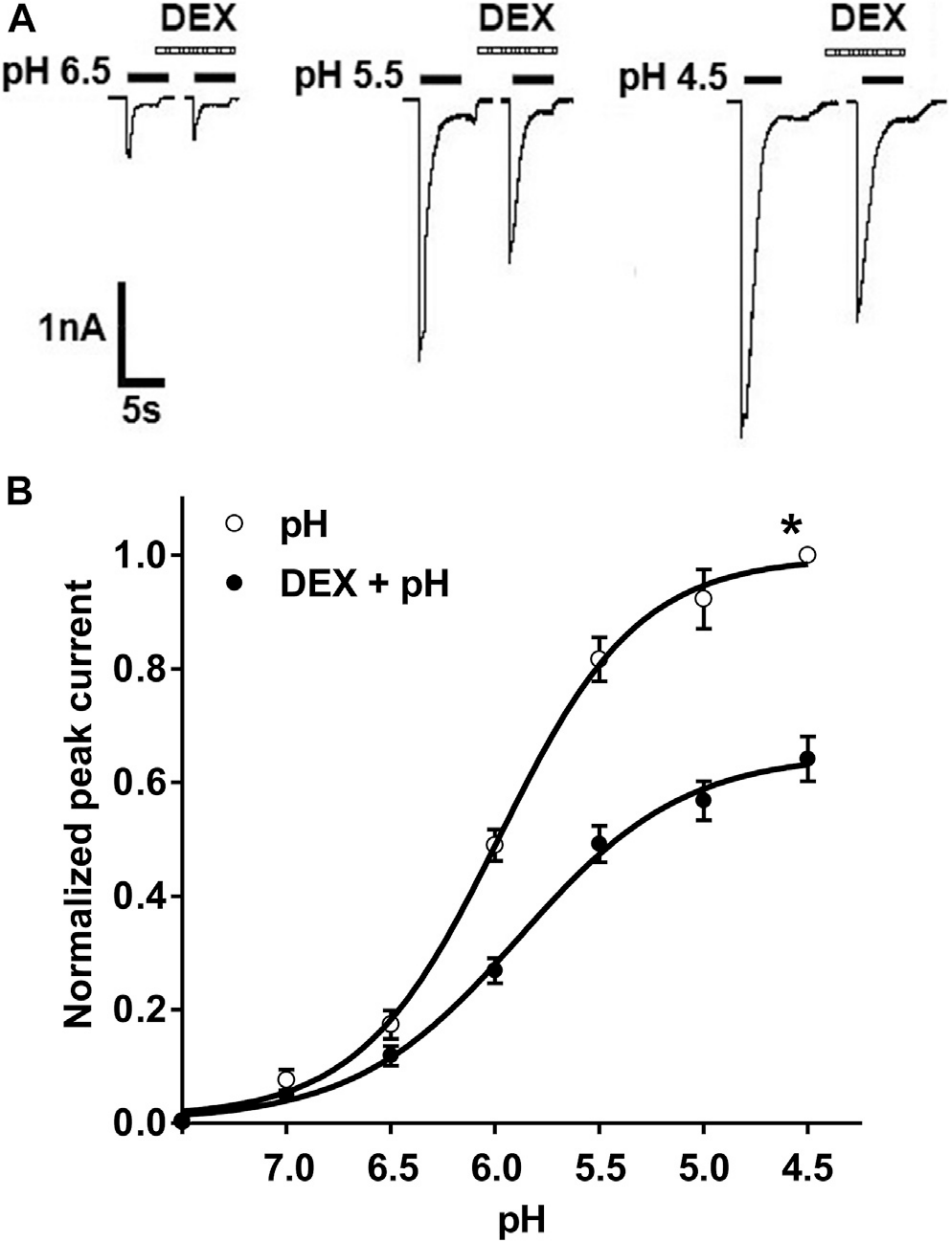
DEX shifts downwards the concentration–response curve for protons. **(A)**. Sequential currents were evoked by different low pH values in the absence and presence of DEX (3 μM) pretreatment. **(B)**. Proton concentration-response curves for ASIC activation in the absence (○) and presence (●) of extracellular 3 μM DEX. Pre-application of DEX shifted downwards the concentration-response curve for proton. Each point represents the mean ± S.E.M. of 6–10 neurons. All peak current values were normalized to the peak current maximally activated by pH 4.5 applied alone in the absence of DEX (marked with asterisk). The figure shows averaged data fitted with the Hill equation.

We then investigated the effect of DEX on concentration-response curve to protons. Currents were measured by applying a range of different low pH values before and after treatment with DEX. Figure 2A shows that pre-application of DEX (3 μM for 2 min) decreased all peak amplitudes of I_pH6.5_, I_pH5.5_ and I_pH4.5_. Figure 2B shows concentration-response to protons in the absence and presence of DEX (3 μM). First, the maximal current response (I_pH4.5_) of curve decreased 35.83 ± 3.91% after DEX was treated to DRG neurons. Second, the Hill coefficient or slope of two curves had not significant difference (pH: n = 1.23 ± 0.16; DEX + pH: n = 1.16 ± 0.21; *p* > 0.1, post hoc Bonferroni’s test). Third, the pH_0.5_ (pH for half-maximal activation) values of two curves had also no statistical difference (pH: pH_0.5_ = 5.97 ± 0.17; DEX + pH: pH_0.5_ = 5.89 ± 0.20; *p* > 0.1, post hoc Bonferroni’s test). These results indicated that the maximum response to protons was inhibited by DEX, but that no shift in proton sensitivity.

### α_2_-ARs Mediate DEX-Induced Inhibition of ASIC Currents

**FIGURE 3:**
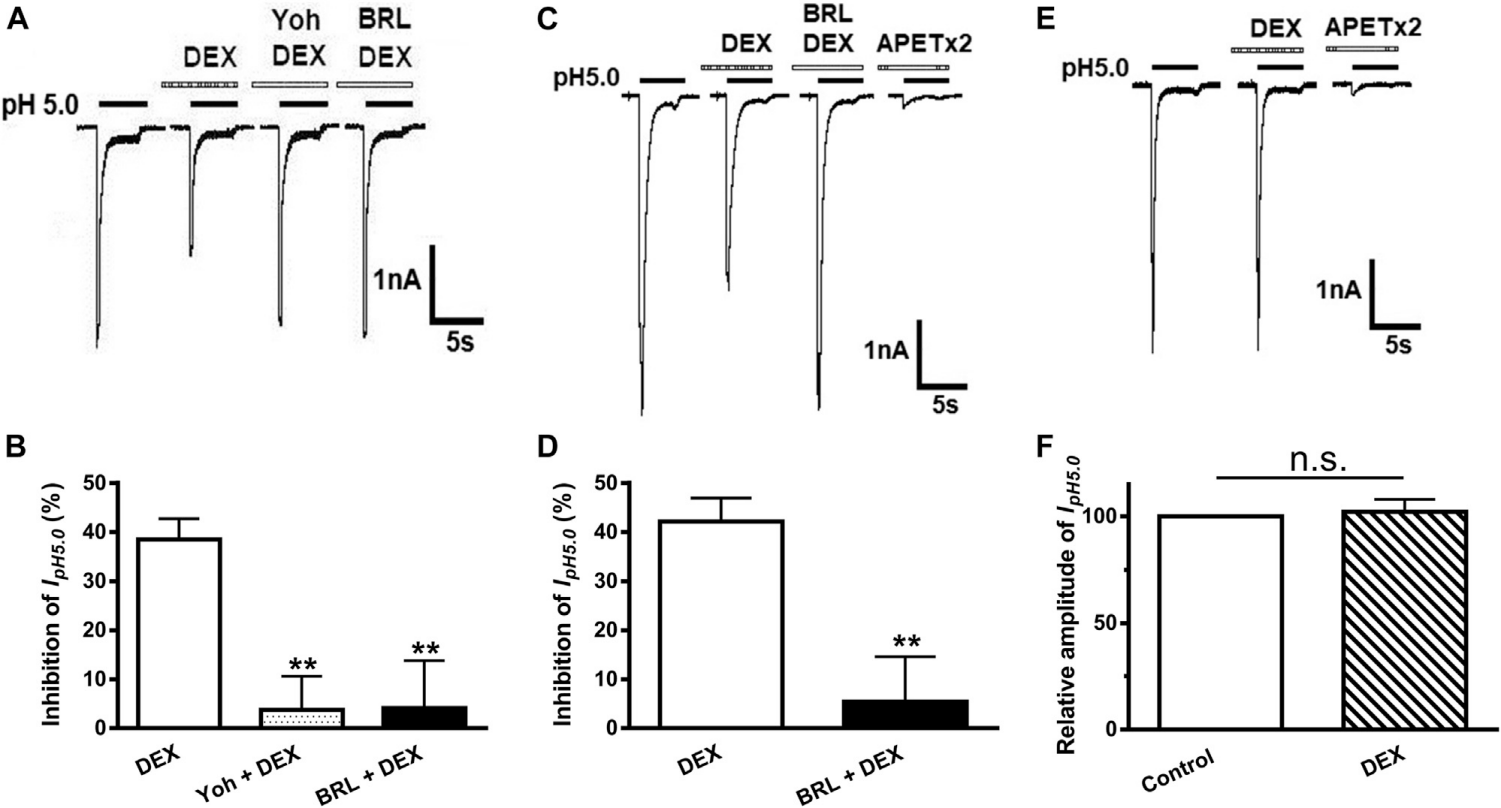
α2-ARs mediate DEX-induced inhibition of ASIC currents in DRG and CHO cells. The current traces in **(A)** and the bar graph in **(B)** show that I_pH5.0_ was inhibited by DEX (3 μM) pre-applied alone for 2 min in DRG neurons, and DEX inhibition of I_pH5.0_ was blocked by the co-application of α_2_-AR antagonist yohimbine (3 μM) or α_2A_-AR antagonist BRL44408 (3 μM). The current traces in **(C)** and the bar graph in **(D)** show that I_pH5.0_ was inhibited by DEX (3 μM) pre-applied alone for 2 min in CHO cells co-expressing ASIC3 and α_2A_-ARs. This inhibiting effect was blocked by the coapplication of α_2A_-AR antagonist BRL44408 (3 μM). The current traces in **(E)** and the bar graph in **(F)** show that DEX (3 μM) had no effect on the I_pH5.0_ in CHO cells expressing ASIC3 alone, but not expressing α_2A_-ARs. Currents were normalized to the control (100%, whitecolumn). Statistical tests were performed using one-way ANOVA followed by *post hoc* Bonferroni’s test, and significance is shown **p < 0.01, n. s, not significant. n = 6 in each column.

DEX is a selective α_2_-AR agonist. To address whether inhibition of ASIC currents by DEX application was mediated by α_2_-ARs, we examined the effect of yohimbine, an α_2_-AR antagonist, on inhibitory effects of DEX on ASIC currents. As shown in Figures 3A,B, DEX-induced suppression of I_pH5.0_ was significantly blocked by yohimbine. The amplitude of I_pH5.0_ decreased 38.48 ± 4.25% by 3 μM DEX pre-treatment alone. In contrast, the amplitude of I_pH5.0_ decreased only 3.73 ± 3.23% in DRG neurons pre-treated with both 3 μM yohimbine and 3 μM DEX (*p* < 0.01, compared with DEX pretreatment alone, one-way ANOVA followed by post hoc Bonferroni’s test, n = 7). Moreover, we also examined the effect of BRL44408, an α_2A_-AR antagonist, on DEX-induced inhibition of ASIC currents. Pre-incubation of BRL44408 (3 μM) alone did not affect the amplitude of I_pH5.0_, but significantly blocked DEX-induced suppression of I_pH5.0_ (Figures 3A,B). The results indicated that DEX inhibited ASIC currents mainly through α_2A_-ARs in DRG neurons.

To further verify whether α_2A_-ARs mediate DEX-induced inhibition of acid-evoked currents, α_2A_-ARs were co-expressed with ASIC3 in CHO cells. We recorded ASIC3-mediated acid currents, since they were completely blocked by APETx2 (2 μM). Similar to that observed in DRG neurons, the ASIC3 currents were inhibited by the pre-application of DEX (3 μM) in CHO cells co-expressing ASIC3 and α_2A_-ARs (Figures 3C,D). The DEX-induced inhibition of ASIC3 currents was also blocked by pre-incubation of 3 μM BRL44408, an α_2A_-AR antagonist (Figures 3C,D). In contrast, DEX had no effect on ASIC3 currents at a concentration of 3 μM in CHO cells expressing ASIC3 alone, but not expressing α_2A_-ARs (Figures 3E,F). The pH 5.0 induced ASIC3 channel activation were completely blocked by APETx2 (2 μM) in CHO cells expressing ASIC3 alone. Guanabenz, another α_2A_-AR agonist, is recently found to activate ASIC3 at neutral pH (Callejo et al., 2020). However, we did not observe that DEX had such an effect when applied in CHO cells expressing ASIC3 alone. DEX (3 or 500 μM) failed to induce any membrane currents at neutral pH (n = 8, data not shown). These results further indicated that α_2A_-ARs mediated DEX-induced inhibition of ASIC3 currents.

### G_i/o_-Protein and PKA Signaling Participate in the Inhibition of ASIC Currents by DEX

**FIGURE 4:**
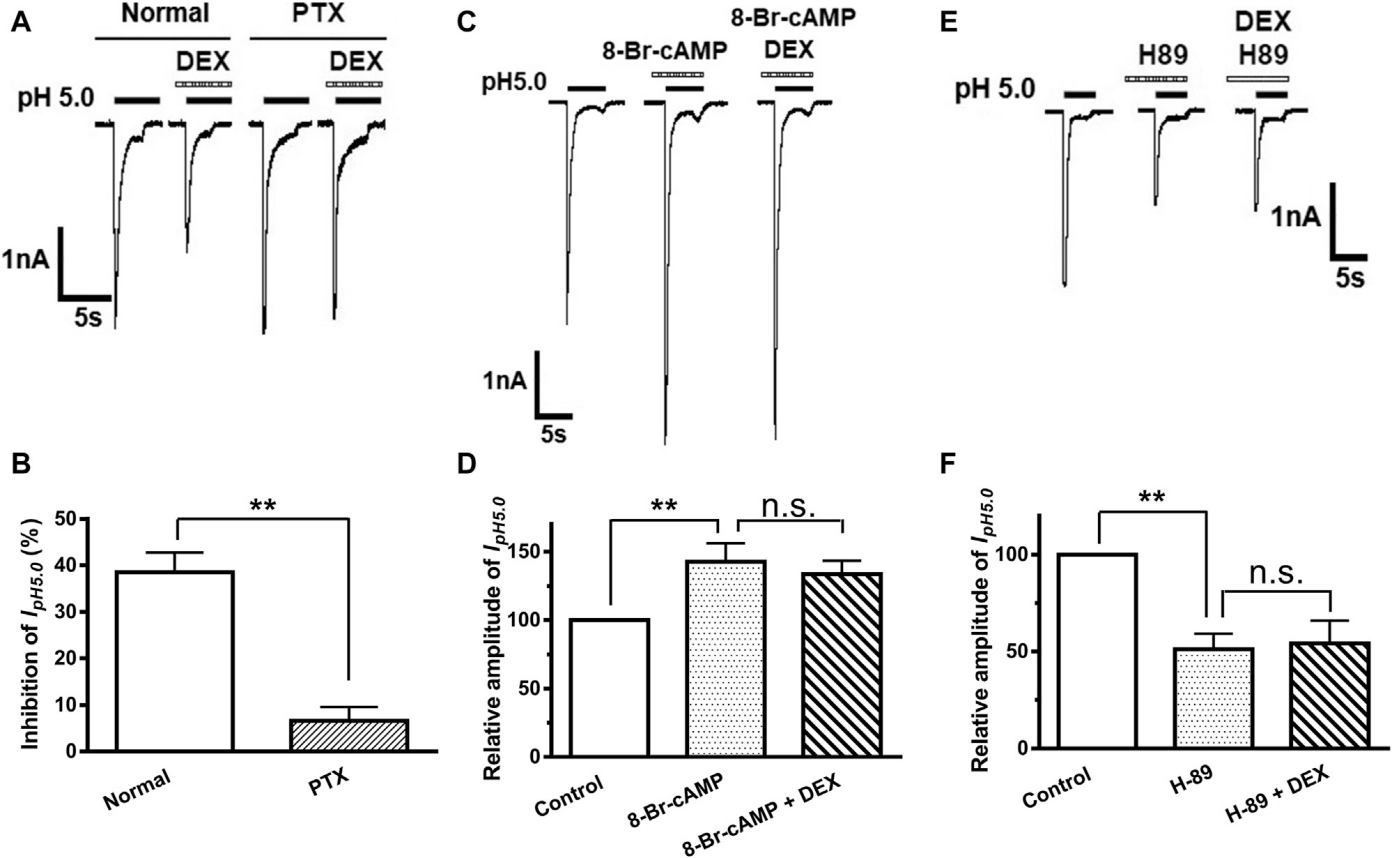
G_i/o_-protein and PKA signaling participate in DEX-induced inhibition of ASIC currents. The current traces in **(A)** and the bar graph in **(B)** show the effects of DEX (3 μM) on I_pH5.0_ in recording pipettes filled with normal and PTX (1 μg/ml) containing internal solution conditions. The current traces in **(C)** and the bar graph in **(D)** show the enhancement of I_pH5.0_ by pre-incubation 8-Br-cAMP (1 mM), a membrane permeable cAMP analog. DEX (3 μM) failed to cause inhibition of I_pH5.0_ in the presence of 8-Br-cAMP (1 mM). The current traces in **(E)** and the bar graph in **(F)** show inhibition of I_pH5.0_ by pre-incubation PKA inhibitor H-89 (0.3 μM). DEX (3 μM) did not cause a further inhibition of I_pH5.0_ in the presence of H-89 (0.3 μM). Statistical tests were performed using one-way ANOVA followed by *post hoc* Bonferroni’s test, and significance is shown **p < 0.01, n. s, not significant. n = 7 in each column.

α_2A_-ARs belong to G_i/o_ protein-coupled receptor family, which leads to a cascade of events, such as inhibition of adenylate cyclase (AC) activity and the cAMP/PKA pathway (Wu et al., 1988). Therefore, we first examined whether DEX-induced inhibition of ASIC currents occurs via G_i/o_ proteins. Pertussis toxin (PTX, 1 μg/ml), an inhibitor of G_i/o_-proteins, was applied internally to DRG neurons and significantly prevented the decrease of I_pH5.0_ amplitude induced by DEX (Figures 4A,B). To further explore intracellular signal transduction mechanisms underlying suppression of ASIC currents by DEX, we observed the effect of 8-Br-cAMP, a membrane permeable cAMP analog, on DEX suppression of ASIC currents. When seven DRG neurons were pre-treated with 8-Br-cAMP (1 mM) for 5 min, the relative amplitudes of I_pH5.0_ were increased to 142.46 ± 13.64% of control. In the presence of 8-Br-cAMP (1 mM), pre-application of DEX (3 μM for 2 min) produced only decreases of 6.25 ± 4.38% on I_pH5.0_ (*p* > 0.1, compared with 8-Br-cAMP treatment alone, one-way ANOVA followed by post hoc Bonferroni’s test, n = 7; Figures 4C,D). We then observed the effect of H-89, a membrane-permeable inhibitor of PKA, on DEX suppression of ASIC currents. When DRG neurons were pre-treated with H-89 (0.3 μM) for 5 min, the relative amplitudes of I_pH5.0_ were reduced to 51.21 ± 7.95% of control (n = 7), suggesting that H-89 mimicked the action of DEX on I_pH5.0_ (Figures 4E,F). In the presence of H-89 (0.3 μM), pre-application of DEX (3 μM for 2 min) no longer further inhibited I_pH5.0_ (*p* > 0.1, compared with H-89 treatment alone, one-way ANOVA followed by post hoc Bonferroni’s test, n = 7; Figures 4E,F). Together, the results indicated that the suppression of ASIC currents by DEX was dependent upon G_i/o_ proteins and intracellular cAMP/PKA signaling pathway.

### DEX Suppresses Acid-Evoked Action Potentials in Rat DRG Neurons

**FIGURE 5:**
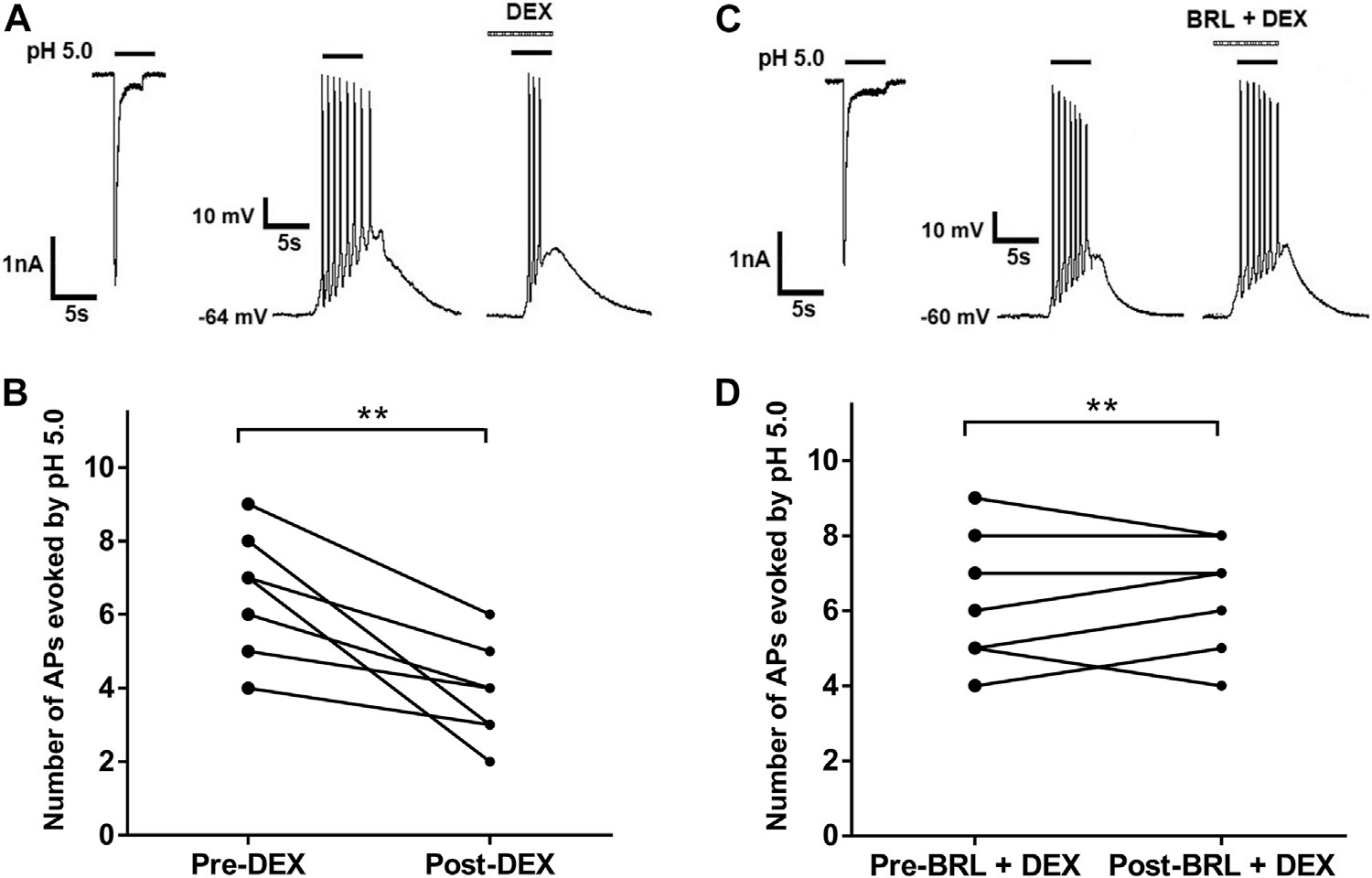
DEX suppresses acid-evoked action potentials in rat DRG neurons. **(A,C)**. In the same DRG neuron, pH 5.0 induced not only an inward current with voltage-clamp recording, but also action potentials with current-clamp recording. Original action potentials were recorded in two DRG neurons before and after application of DEX (3 μM) alone **(A)** or coapplication of both DEX (3 μM) and BRL44408(BRL, 3 μM) for 2min **(C)**. AMG9810 (5 μM) was used to block proton-induced TRPV1 activation. **(B,D)**. The graphs show the number of pH 5.0 acid-evoked action potentials decreased by pre-application of DEX alone, but not by coapplication of both DEX and BRL44408. **p < 0.01, paired *t*-test, n = 7 cells.

ASICs are trimeric cation-permeable channels. Once activation of ASICs by protons causes cation influx (largely Na^+^) and membrane potential depolarization, resulting in bursts of action potentials if the proton-induced depolarization is large enough to activate Na_v_ subunits (Pattison et al., 2019). We further observed whether DEX had effects on acid-evoked action potentials of rat DRG neurons. Although proton-induced TRPV1 activation was blocked in the presence of 5-μM AMG9810, we observed that an acid stimulus of pH 5.0 could trigger bursts of action potentials (APs) in DRG neurons under current-clamp conditions (Figures 5A,C). Consistent with that observed under voltage-clamp conditions, DEX also decreased the number of APs evoked by acidic stimuli of pH 5.0 in DRG neurons (Figures 5A,B). In seven DRG neurons with DEX (3 μM for 2 min) pre-treatment, the number of APs evoked by acidic stimuli of pH 5.0 significantly decreased (*p* < 0.01, paired *t*-test, n = 7, Figure 5B). However, the number of APs had not changed in other seven DRG cells co-treated with both BRL44408(BRL, 3 μM) and DEX (3 μM) (*p* > 0. 1, paired *t*-test; n = 7, Figures 5C,D). These results indicated that DEX also suppressed acid-evoked action potentials in rat DRG neurons through α_2A_-ARs.

### DEX Relieves Acid-Induced Nociceptive Behaviors in Rats

**FIGURE 6:**
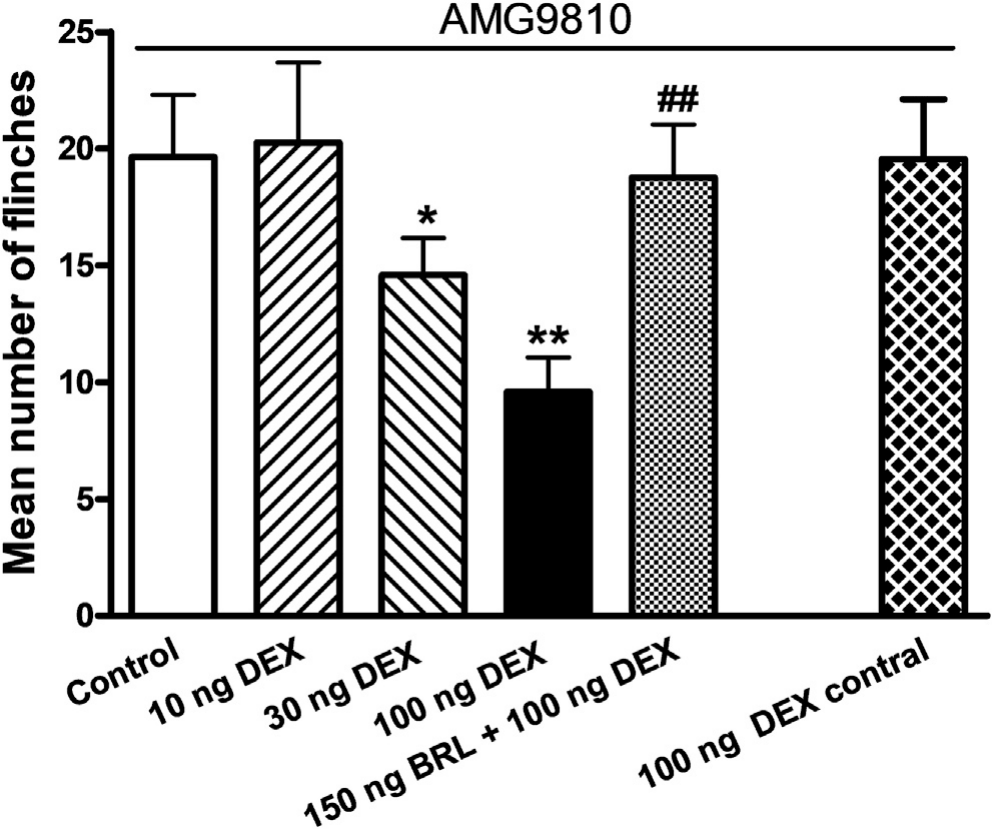
DEX relieves acid-induced nociceptive behaviors in rats. In the presence of the TRPV1 inhibitor AMG9810 (10 μM), nociceptive responses were evoked by intraplantar injection of acetic acid (1%, 50 μl) in each rat. Intraplantar pretreatment of DEX (10, 30 and 100 ng) dose-dependently reduced the number of acid-induced flinching. DEX (100 ng) inhibition of acid-induced nociceptive behaviors is reversed by co-injection of α_2A_-AR antagonist BRL44408 (BRL, 150 ng) so that behaviors in these rats were no different from the control rats treated with acetic acid alone. Rats injected with acetic acid in one hindpaw and DEX into the contralateral hindpaw (100 ng contral) had nociceptive behaviors similar to those seen in control rats. Each bar represents the number of flinches that animals spent licking/lifting the injected paw during first 5 min observation period. **p < 0.01, one-way ANOVA followed by *post hoc* Bonferroni’s test, compared with control column; ##*p* < 0.01, one-way ANOVA followed by *post hoc* Bonferroni’s test, compared with 100 ng DEX column. Each group represents the mean ± S.E.M. of 10 rats.

Above results demonstrated that DEX inhibited ASIC activity *in vitro*. We further ascertained whether DEX had effect on ASIC-mediated nociceptive behaviors through interacting with ASICs *in vivo*. After acetic acid was injected into rat hind paws, rats displayed an intense flinch/shaking response even if AMG 9810 (10 μM) blocked the activation of TRPV1. We found that pretreatment with DEX (10, 30, and 100 ng) dose-dependently relieved the acid-induced nociceptive behaviors (*p* < 0.05 and 0.01, one-way ANOVA followed by post hoc Bonferroni’s test, n = 10; Figure 6). However, Co-injection of BRL44408 (BRL, 150 ng) blocked the relieving effect of 100 ng DEX on acid-induced nociceptive behaviors. The mean number of flinches in these rats significantly increased, compared with that observed in rats pretreated with 100 ng DEX alone (*p* < 0.01, one-way ANOVA followed by post hoc Bonferroni’s test, n = 10; Figure 6), and were no different from control rats without DEX pretreatment. In addition, injection of 100 ng DEX into the contralateral paws did not relieved acid-induced nociceptive behaviors. These results indicated that DEX relieved acid-induced nociceptive behaviors in rats through activation of α_2A_-ARs localized in the injected hindpaw.

## Discussion

The present data demonstrated that selective α_2_-AR agonist DEX inhibited the electrophysiological activity of ASICs through α_2A_-ARs. DEX reduced ASIC-mediated and acid-evoked currents and action potentials in dissociated rat DRG neurons. PTX-sensitive G_i/o_ proteins and cAMP/PKA signaling cascade was involved in the inhibition of ASICs by DEX. Behaviorally, DEX also relieved acid-induced nociceptive responses in rats by activating peripheral α_2A_-ARs.

It has been found that six ASIC subunits are expressed in neuron cell bodies in DRG, and ASIC3 subunit is the most abundant (Deval et al., 2008). In the presence of AMG9810, the low pH-evoked currents were mediated by ASICs in rat DRG cells, since they were completely blocked by amiloride and APETx2. The present study showed that DEX, a selective α_2_-AR agonist, inhibited ASIC currents and the number of action potentials evoked by low pH stimulation. Moreover, DEX accelerated the inactivation of ASICs, which also helps to reduce the number of action potentials evoked by acidic stimuli. DEX inhibited the electrophysiological activity of ASICs in rat DRG neurons through α_2_-ARs. First, another α_2_-AR agonist clonidine has also been shown to effectively inhibit ASIC currents. Second, DEX-induced inhibition was completely blocked by the α_2A_-AR antagonist BRL44408. Last, DEX could also inhibit acid-evoked currents mediated by ASIC3 in CHO cells co-expressing α_2A_-ARs and ASIC3, but not in CHO cells only expressed ASIC3 and lacked α_2A_-AR expression, further suggesting that the inhibition of ASIC3 currents by DEX required the co-expression of α_2A_-ARs. In addition, only some, but not all, ASIC-like responses were inhibited by DEX/clonidine, which may be related to the degree of the co-expression of α_2A_-ARs and ASIC3 in rat DRG neurons. Thus, we speculated that DEX-induced inhibition of ASIC currents maybe also occur only in DRG neurons co-expressing α_2A_-ARs and ASICs, although the direct evidence needs to further determine. It has been shown that three subtypes of the α2-ARs (α_2A_-, α_2B_- and α_2C_-ARs) are expressed in the rat DRGs (Shi et al., 2000). But α_2B_-AR subtype is found only in a very small population (Chung et al., 1993; Shi et al., 2000). The expression of α_2A_ and α_2C_-AR subtypes is regulated by peripheral nerve injury. Proteins and mRNA levels of α_2A_ and α_2C_-AR subtypes are increased and decreased, respectively, in DRG neurons after chronic constriction injury of sciatic nerve (Cho et al., 1997; Shi et al., 2000). In addition to ASICs, other cation channels, such as Nav1.8, TRPV1 and TRPM8 are also inhibited via α_2A_-AR subtype in DRG neurons (Bavencoffe et al., 2010; Gu et al., 2015; Lee et al., 2020). In addition, α_2A_-AR agonist is recently found to activate ASIC3 at neutral pH and potentiate acid-gated currents (Callejo et al., 2020). However, we observed that DEX failed to induce any membrane currents at neutral pH when applied in CHO cells expressing ASIC3 alone. The lack of such a role of DEX may be due to the difference of the molecular backbone between DEX and guanabenz, although both of them are α_2A_-AR agonists.

α_2A_-AR is a G_i/o_ protein-coupled receptor that inhibits AC and reduces cAMP production upon activation (Wu et al., 1988). The present data demonstrated that G_i/o_ proteins and intracellular cAMP/PKA signal transduction were involved in DEX-induced inhibition of ASIC currents in rat DRG neurons. First, preventing G_i/o_ recruitment by PTX treatment blocked DEX-induced inhibition of ASIC currents. Second, DEX-induced inhibition was lack after DRG neurons treated with the cAMP analog 8Br-cAMP. Last, inhibition of intracellular PKA with H-89 mimicked the inhibiting effect of DEX on ASIC currents. Then on the base of these, DEX no longer further inhibited ASIC currents. These findings were consistent with the general notion that stimulation of α_2A_-ARs by DEX brings about G_i/o_-mediated inhibition of AC and reduction of intracellular cAMP levels, suggesting that DEX inhibited ASIC currents through PTX-sensitive Gi/o proteins and classical AC/cAMP/PKA signaling pathway. Previous studies show that ASICs are modulated by intracellular cAMP/PKA signaling. For example, inhibition of PKA phosphorylation of ASICs decreases the amplitude of ASIC currents in cortical neurons (Chai et al., 2007). It has been shown that cAMP-dependent signaling pathway involves in the inhibition of ASIC-mediated functional activity by sumatriptan, a G_i/o_ protein-coupled 5-HT_1D_ receptor agonist, in rat trigeminal ganglion neurons (Guo et al., 2018). Our previous studies found that activation of μ-opioid receptors or CB1 cannabinoid receptors inhibits ASICs in DRG neurons in a cAMP/PKA-dependent manner (Liu et al., 2012; Cai et al., 2014). These studies provide further support that the suppression of ASIC currents by DEX was dependent upon intracellular cAMP and PKA signaling pathway.

Intraplantar injection of low pH solution produces an intense flinch/shaking response in rats by activating ASICs (Deval et al., 2008; Omori et al., 2008). In the presence of AMG9810, ASICs are activated by injected protons, causing cation influx (largely Na^+^) and membrane depolarization in nociceptors, where nociception will occur if the depolarization is large enough to activate Na_v_ subunits, resulting in generation of action potential that transmit nociceptive signals (Pattison et al., 2019). The present data show that peripheral pretreatment of the DEX relieved the ASIC-mediated nociceptive behaviors in a dose-dependent manner. The DEX′ actions occurred locally rather than systematically, since the low pH- evoked nociceptive behaviors did not changed when DEX was injected into contralateral hindpaws. Local application of BRL44408, a specific α_2A_-AR antagonist, significantly blocked the effect of DEX on ASIC-mediated nociceptive behaviors, suggesting that the DEX exerted its analgesic effect by acting directly on α_2A_-AR subtypes localized on nociceptors. It has been shown that anti-noceptive effects of DEX are mainly mediated via α_2A_-AR subtype, since the effects are absent in α_2A_-AR mutant mice, but intact in α_2B_- and α_2C_-AR mutant mice (Malmberg et al., 2001). Obviously, the behavioral data corroborated the electrophysiological studies and vice versa. In this work, we used cell bodies of DRG neurons as a simple and accessible model to examine the characteristics of the membrane of peripheral terminals. Inhibition of ASICs by DEX may also occur in peripheral terminals. So it's also possible that DEX relieved low pH- evoked nociceptive behaviors by inhibiting ASICs localized in nociceptors, at least partially. DEX activated α_2A_-ARs and then decreased ASIC-mediated currents and action potentials in primary sensory neurons, resulting in reduction of ASIC-mediated pain.

Protons are released and result in a decrease in pH value in some pathological pain conditions, such as postoperative pain. The extracellular decreased pH is sustained for a few days after incision (Woo et al., 2004). Peripheral ASIC3-containing channels have been found to detect local changes in pH and contribute to postoperative pain (Deval et al., 2011). Intra-operative application of an ASIC3 inhibitor has a potent analgesic effect on postoperative pain (Deval et al., 2011). Clinically, intra-operative DEX decreases requirements for postoperative analgesics of various surgical patients, improves pain management in post-anesthesia care unit, and has no obvious side effects (Alhashemi and Kaki, 2004; Arain et al., 2004). The present results that DEX-induced inhibition of ASICs in primary sensory neurons provided a clue that ASICs may be therapeutic targets for peripheral DEX to relieve some types of pain, such as postoperative pain.

## Conclusion

In summary, our results suggested DEX-induced inhibition of ASIC-mediated the electrophysiological activity and pain, revealing a novel peripheral mechanism underlying DEX analgesia. It is clinically important that local application of DEX can not only effectively relieve pain by inhibiting ASICs in primary sensory neurons, but also this route of administration would avoid side-effects that accompany systemic activation of α_2A_-ARs.

## Data Availability

The raw data supporting the conclusions of this article will be made available by the authors, without undue reservation

## Abbreviations

α2-AR, α2 adrenergic receptor; AC, adenylate cyclase; ANOVA, analysis of variance; APs, action potentials; ASIC, acid-sensing ion channels; CHO, Chinese hamster ovary; DEX, dexmedetomidine; DRG, dorsal root ganglion; IpH, proton-gated current; PKA, protein kinase A; PTX, pertussis toxin; TRPV1, transient receptor potential vanilloid receptor-1.
